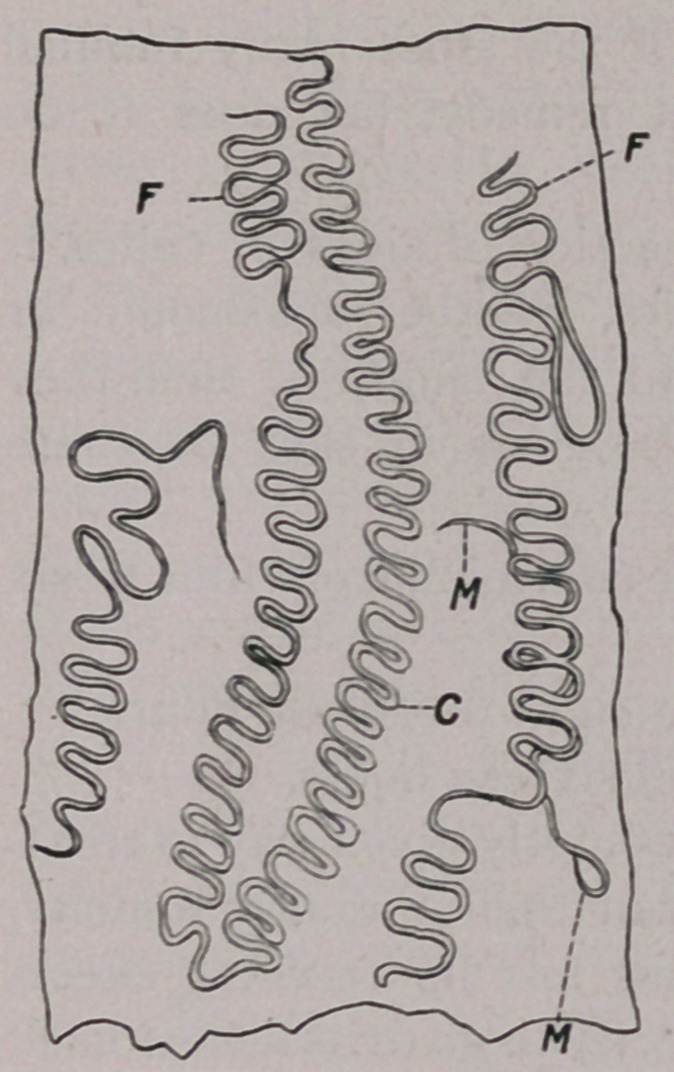# Notes on Parasites*Preliminary note on *Myzomimus* gen. nov., type species *M. scutatus*, Mueller, ’69, a parasite in cattle.

**Published:** 1892-02

**Authors:** Charles W. Stiles

**Affiliations:** Bureau of Animal Industry, U. S. Department of Agriculture


### THE JOURNAL
OF
### COMPARATIVE MEDICINE AND VETERINARY ARCHIVES.

Vol. XIII. FEBRUARY, 1892. No. 2.

### NOTES ON PARASITES.*

Charles W. Stiles, Ph. D.

In 1869 Prof. Mueller, of Vienna, found a curious threadworm in the œsophagi of five or six Polish and Hungarian cattle and in the œsophagus of an old horse, and described the same as *Spiroptera scutata œsophagea bovis*, giving at the time a short diagnosis of the animal in Latin. The same parasite is reported by Harms and Zürn from the œsophagi of sheep, and by Korzil from swine.

All of these observations were made in Europe, and the various authors have added scarcely anything to the data given by Mueller.

At Washington, D. C., this parasite is quite frequent, and has been observed by Dr. Curtice and Dr. Hassall as well as by myself. Upon studying the helminth, I have found several anatomical characteristics which scarcely agree with the description given in most text-books at present, and furthermore, I am convinced that the anatomical characters which are described below are sufficient to exclude the parasite in question from the genera *Filaria* and *Spiroptera* in which it is generally placed, so that I have, although somewhat reluctantly, created a new genus for it under the name *Myzomimus*.

Myzomimus gen. nov. Stiles, 1892. Diagnosis Long filiform worms, of nearly uniform thickness throughout the entire length, except at the head and tail, where they are slightly attenuated. Type specimen (*M. scutatus*), inhabiting the epithelium of the œsophagus in cattle. Head slightly compressed laterally, tail

*Preliminary note on *Myzomimus* gen. nov., type species *M. scutatus*, Mueller, '69, a parasite in cattle.

(male) compressed dorso-ventrally. Anterior portion of body beset for 1–3 mm., with shield-like differentiations of the cuticle, and with side membranes (wings or folds) ; mouth small, unarmed, oblong dorso-ventrally; lips absent; two minute oral papillæ, placed laterally. In the median lines, directly back of mouth, are two semi-lunar sucker-like depressions, hence the name Myzomimus, from the Latinized Greek, meaning something which imitates or resembles a sucker. Two very large circular cervical papillæ, cuticle finely annulated. Œsophagus divided into two portions *Male* smaller than female, with tail curved ventrally; *six* pairs of præ-anal papillæ and 4-6 pairs of post-anal papillæ ; lateral folds of tail asymmetrical; anus in front of tip of tail; right spicule short and stout; left spicule very long and slender; one testis present *Female*, vulva in posterior portion of the body; vagina long ; uterus double.

Species *Myzomimus scutata*, Mueller, 1869. The following may be added to the characters given above. The shields are arranged nearly symmetrically in the four sub-median fields; generally a continuous or interrupted row of shields is found in each field, together with other shield in various positions. Rings of the cuticle 0.008 mm. broad. Side membranes do not extend far beyond the shielded portion, and are interrupted or broken in several places. *Male* 4-5 cm. long. Œsophagus, 1st portion 0.67 mm., 2nd portion 4.6 mm. long. Tail curled ventrally ; anus 0.36 mm. from tip ; 6 pairs of præ-anal, and 4-6 pairs of post-anal papillæ. An unpaired dorsal ridge supports the left fold of the tail. *Female* 8-11 cm. long. Œsophagus, 1st portion 0.85 mm. long, 2nd portion 7.8 mm. long. Vulva 4.5 mm. from the tip of tail. Vagina extremely long. Ovoviviparous. Embryo provided with a boring apparatus. All the above measurements are variable.

My reasons for creating a new genus are the following : The dorsal and ventral sucker-like depressions on the head do not occur in any known genus of the Filaridæ ; the same may be said of the shields; the vulva in the genus *Filaria* is in the anterior portion of the body near the mouth, so our species is excluded from that genus ; the genus *Spiroptera* is very indistinctly separated from the genus Filaria, but in Spiroptera, too, the vulva is the anterior portion of the body ; our species cannot enter the genus *Dispharagus*, since a single ovary is supposed to obtain in members of that group, and it is excluded from the genus *Hystrichis* by the absence of spines on the head. The other genera of the family *Filaridæ* are so totally different from the species under consideration, that I need not discuss them.

The accompanying illustration was drawn from nature by Mr. Hains, one of the artists of the Bureau. It shows three worms in a portion of the œsophagus. It will be seen from the figure that the worms bore small canals in the epithelium. F. female, M. male alongside of the female, C. portion of the canal formerly occupied by the parasite.

A complete description of *M. scutatus*, with its pathological effect and so much of its biology as has been made out, will appear later in the Report of this Bureau.

Bureau of Animal Industry,
U. S. Department of Agriculture, January 5, 1892.

## Figures and Tables

**Figure f1:**